# Assessing causality between mitochondrial-associated proteins with musculoskeletal diseases: A Mendelian randomization study

**DOI:** 10.1097/MD.0000000000041731

**Published:** 2025-03-07

**Authors:** Jia-Chen Li, Wei-Sheng Huang, Da-Hang Yang, Qi-Fei He, Wei Sun

**Affiliations:** a Department of Orthopedics, Shenzhen Second People’s Hospital/First Affiliated Hospital of Shenzhen University, Shenzhen, Guangdong, China; b Shantou University Medical College, Shantou, China.

**Keywords:** ankylosing spondylitis, Mendelian randomization, mitochondria, musculoskeletal disease, osteoarthritis, osteoporosis, rheumatoid arthritis

## Abstract

Musculoskeletal diseases are the leading cause of disability-adjusted life years. Mitochondria, often referred to as the “powerhouses” of cells, are believed to play a role in regulating cellular metabolism and differentiation, potentially influencing the occurrence and progression of musculoskeletal diseases. However, the exact causal relationships remain to be defined. This study aimed to investigate the causal relationships between mitochondrial biological functions and musculoskeletal diseases (including osteoarthritis (OA), osteoporosis, rheumatoid arthritis (RA), and ankylosing spondylitis through Mendelian randomization (MR) analysis). We systematically summarized data related to mitochondrial functional proteins and musculoskeletal diseases from the IEU OpenGWAS and UK Biobank databases. We used single nucleotide polymorphisms significantly associated with musculoskeletal diseases as instrumental variables. The inverse variance weighting method performed the main MR analysis. We used Mendelian randomized residual sum of pleiotropy and outliers, MR-Egger regression, Cochran *Q* statistic, Rucker *Q* statistic, Radial-MR, weighted median, simple mode, weighted mode, and leave-one-out analysis methods as supplementary analyses. First, 14 positive mitochondrial functional proteins were screened out. After Bonferroni correction, COA3 and COX4I2 were found to be causally related to OA and act as protective factors. We identified a causal relationship between SLC25A18 and RA as a risk factor. This study provides genetic support and offers new evidence regarding the roles of COA3, COX4I2, and SLC25A18 in the pathophysiology of OA and RA. This study paves the way for a deeper understanding of the pathological mechanisms of musculoskeletal diseases and provides information for their prevention strategies and treatments.

## 1. Introduction

Musculoskeletal diseases encompass a spectrum of chronic conditions affecting the bones, cartilage, joints, muscles, and spine^[1,2]^ and mainly include^[3]^ osteoarthritis (OA), osteoporosis (OP), rheumatoid arthritis (RA), and ankylosing spondylitis (AS). Approximately 1.71 billion people worldwide suffer from musculoskeletal diseases.^[4]^ It is the largest factor leading to global disability-adjusted life years, accounting for about 149 million, which constitutes 17% of the total global burden of global disability-adjusted life years. It is the main cause of adults’ loss of independence and reduced quality of life.^[5]^ The current mainstream treatments include drug treatment, surgical treatment, biological targeted therapy, and traditional Chinese medicine physiotherapy. However, these methods often fail to achieve the desired treatment results.^[6]^ Hence, further research into the etiology and pathogenesis of musculoskeletal diseases, along with the identification of novel treatment targets, is of great significance for the development of effective treatment strategies.

Mitochondria, characterized by double-membrane structure, are essential intracellular organelles responsible for the synthesis of adenosine triphosphate (ATP), which serves as the principal energy carrier for various physiological and biochemical activities within living cells.^[7]^ Mitochondria serve as the powerhouse of eukaryotic cells, regulating cellular metabolism and differentiation.^[8]^ Some studies have found that mitochondrial dysfunction may lead to intracellular disorder or cell dysfunction, disrupt the balance of osteoblast and osteoclast activity, lead to premature bone aging, and ultimately affect the stability of the musculoskeletal system. Furthermore, oxidative stress in mitochondria raises the production of reactive oxygen species, which leads to redox imbalance and changes in cellular physiology that make OA, AS, RA, and other diseases more likely to happen.^[9–13]^ However, some studies believe that transient mitochondrial inhibition may serve as a cellular protective strategy, aiding cell survival during decreased energy demand.^[14,15]^ Despite the continuous efforts of researchers, a comprehensive theoretical framework has not yet been established to elucidate the causal relationship between mitochondrial function and musculoskeletal diseases.

Mendelian randomization (MR)^[16]^ is an epidemiological technique that employs genetic variation as an instrumental variable (IV) to evaluate the causal relationship between exposure and outcome. The principle is that genetic variation is inherited randomly at conception. This inherent randomness grants MR the advantage of effectively mitigating confounding effects and resolving reverse causation in epidemiological studies.^[17]^ In this study, our objective was to access the causal relationship between mitochondrial-associated proteins and musculoskeletal diseases through MR analysis based on genome-wide association study (GWAS) data.

## 2. Materials and methods

### 2.1. Study design

**Figure 1.**
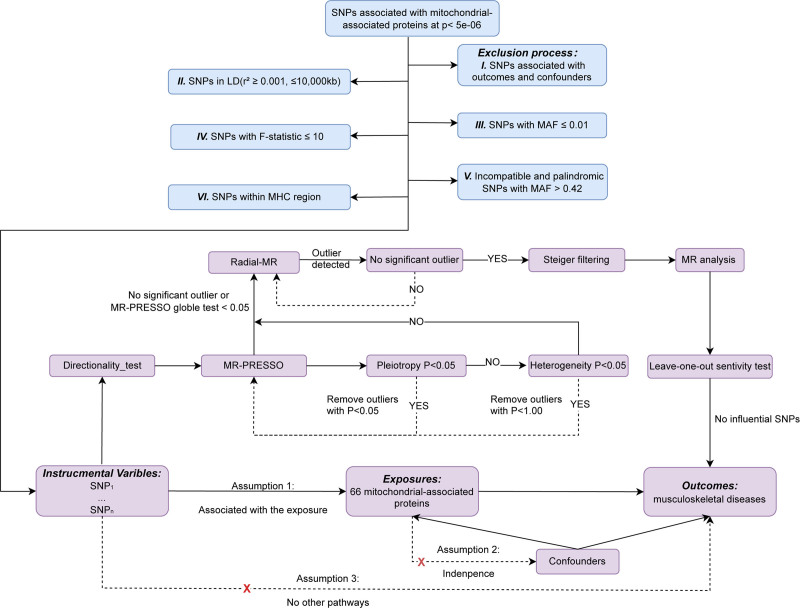
Flowchart showing the discharge standards of SNPs and the principle of MR. MR = Mendelian randomization, SNP = single nucleotide polymorphisms.

The study adhered to the most recent STROBE-MR guidelines for doing MR analysis based on 3 key presumptions: 1. Instrumental variables are closely associated with mitochondrial proteins. 2. Any potential confounding factors do not influence the chosen instrumental variables. 3. The chosen instrumental variables are unrelated to the outcome but related to the exposure. Additionally, meeting other assumptions requires the absence of statistical interactions.^[18]^ Figure 1 illustrates the schematic representation of the methodology used in MR analysis.

### 2.2. Data selection

We used IEU OpenGWAS data related to mitochondrial biological functions as exposure variables (Table S1, Supplemental Digital Content, http://links.lww.com/MD/O462). Single nucleotide polymorphisms (SNPs) meeting a genome-wide significance threshold (*P* < 5 × 10^−6^) were used as possible instrumental variables^[19]^ to find genetic loci linked with these proteins. As shown in Table 1, GWAS summary data for musculoskeletal diseases, including OA, OP, RA, and AS, were obtained from the UK Biobank Consortium and are also accessible through the IEU OpenGWAS website.

**Table 1:** GWAS data pertaining to and musculoskeletal diseases.

GWAS ID	Year	Trait	Consortium	Sample size	Number of SNPs	Population	Author
ukb-b-14486	2018	Osteoarthritis	MRC-IEU	462,933	9851,867	European	Ben Elsworth
ukb-a-87	2017	Osteoporosis	Neale Lab	337,159	10,894,596	European	Neale
ukb-d-M06	2018	Rheumatoid arthritis	Neale Lab	361,194	9944,222	European	Neale
ukb-a-88	2017	Ankylosing spondylitis	Neale Lab	337,159	10,894,596	European	Neale

### 2.3. Instrumental variables selection

The inclusion criteria for IVs are as follows (Fig. 1): I. According to the basic assumptions 2 and 3 of MR, SNPs related to outcomes and confounding factors are removed. Using the 1000 Genomes Project European sample data as the reference panel, the calculation of linkage disequilibrium between SNPs revealed an unbalanced distribution. II. Exclude ①. kb ≤ 10,000; ②. *R*^2^ ≥ 0.001. III. Exclude SNPs with a minor allele frequency ≤ 0.01. When palindromic SNPs exist, use allele frequency information to infer the forward strand allele.^[20]^ IV. The *F* statistic was used to assess the IVs’ strength. It was calculated using the following formula: *F* = *R*^2^ × (N − 1 − *K*)/((1 − *R*^2^) × *K*), where *R*^2^ represents the percentage of exposure variance that can be explained by genetic variation, *N* is the sample size, and *K* is the number of instruments. SNPs with *F* statistics ≤ 10 should be excluded. The *F* statistic >10 indicates the absence of significant weak instrument bias.^[21]^ V. To avoid strand direction or allele coding distortion, exclude incompatible palindromic SNPs or those with allele frequencies above 0.42. VI. Additionally, exclude variants found in the extended major histocompatibility complex region because of the region’s high disease association and complex linkage disequilibrium structure, which makes it susceptible to MR assumption violations and horizontal pleiotropy.

### 2.4. Mendelian randomization analysis

Figure 1 shows a flowchart describing the MR analysis. After obtaining the instrumental variables, the directionality-test is initially conducted to detect and remove outliers indicative of reverse causality. The Mendelian randomized residual sum of pleiotropy and outliers (MR-PRESSO) global test identifies outliers (SNPs with *P*-value < .05) and then removes horizontal pleiotropy if present. A *P*-value for the MR-Egger intercept of less than .05 implies horizontal pleiotropy, which is tested using MR-Egger regression. After removing outliers, we run MR-PRESSO a second time. Then, using inverse variance weighting (IVW) and MR-Egger methods, we examined SNP heterogeneity based on the SNPs that remained after pleiotropy correction. The existence of heterogeneity was verified by the statistics Cochran *Q* (for IVW) and Rucker *Q* (for MR-Egger).^[22]^ In this stage, SNPs with *P*-values < 1.00 are removed from the MR-PRESSO analysis if heterogeneity is significant (*P* < .05 for both Cochran *Q* and Rucker *Q* statistics) and horizontal pleiotropy is not observed.^[23]^ Then run MR-PRESSO again. A prerequisite for implementing the Radial-MR method is the presence of at least 5 SNPs. If no SNPs with *P* < 1.00 are found, if significant SNPs are undetected in the MR-PRESSO analysis, or if the global test *P*-value is < .05 during the MR-PRESSO analysis, the Radial-MR is used to further identify and remove outliers with *P*-values < .05.^[24]^ After outlier removal, Radial-MR is repeated until no outliers are identified. Following the aforementioned procedures, SNPs that account for a larger percentage of the variance in the outcome than in the exposure are removed via Steiger filtering if neither horizontal pleiotropy nor heterogeneity are present.^[25]^ Subsequently, the main MR analysis is conducted using the IVW method.^[26]^ Lastly, a “leave-one-out” analysis is performed to find influential SNPs. If these SNPs were absent, we considered the conclusion to be reliable.^[27]^ There are other MR methods available additionally, including MR-Egger, weighted median, simple mode, and weighted mode. All SNPs can be used as invalid instruments based on the MR-Egger method, but variants must meet the InSIDE assumption. In the presence of pleiotropic effects, this method allows for the estimation of appropriate causal effects.^[28]^ An alternative method to get consistent estimates is to employ the weighted median method when fewer than 50% of IVs are invalid and most of IVs do not show directional horizontal pleiotropy.^[29]^ Clusters are generated using simple mode by calculating the causal effects on individual SNPs. The largest SNP cluster’s causal effect estimate was used to calculate the causal effect estimate. The weighted mode method uses the same procedure but grants each SNP a weight.

### 2.5. Sensitivity analysis

In addition, we also conducted other methods to conduct sensitivity analysis to evaluate the robustness of our research results. Debiased inverse-variance weighted method is a simple modification of the IVW estimator,^[30]^ and it eliminates the weak instrument bias of the IVW method and maintains stronger robustness even in the presence of numerous weak instruments. Penalized inverse-variance weighted estimator addresses the issue of weak IVs by adjusting the original IVW estimates using a penalization method,^[31]^ with the variance estimation adjusted to account for the presence of balanced horizontal pleiotropy. Robust adjusted profile score allows for the inclusion of weak instrumental variables,^[32]^ enabling robust statistical estimation of MR through these weak instruments. Considered to be much more efficient than MR-Egger, the constrained maximum likelihood and model averaging-based MR method^[33]^ has been developed for controlling correlated and uncorrelated pleiotropic effects. The mixlE is a novel technique that combines Egger regression and lVW with the objective of preserving each method’s advantages while resolving its main drawbacks.^[34]^ MixIE-MA is the default version, and MixlE-MA-DP is a data perturbation-based version. The uncertainty of weak effects because of polygenicity was taken into account in the Bayesian weighted Mendelian randomization model, and the issue of IV assumption violations brought on by pleiotropy was resolved by identifying outliers through Bayesian weighting.^[35]^

We performed Bonferroni correction to the *P*-value for the IVW method. A *P*-value of <7.58 × 10^−4^ (0.05/66 exposures) was considered to be strong evidence of a causal relationship, while a *P*-value of less than .05 but exceeding 7.58 × 10^−4^ was thought to be potentially significant. All statistical analyses conducted in this study were performed using the “TwoSampleMR,” “MendelianRandomization,” “phenoscanner,” “MRPRESSO” and “RadialMR” packages in R software (4.3.2).^[36]^

## 3. Result

### 3.1. Results of the MR analysis

**Figure 2.**
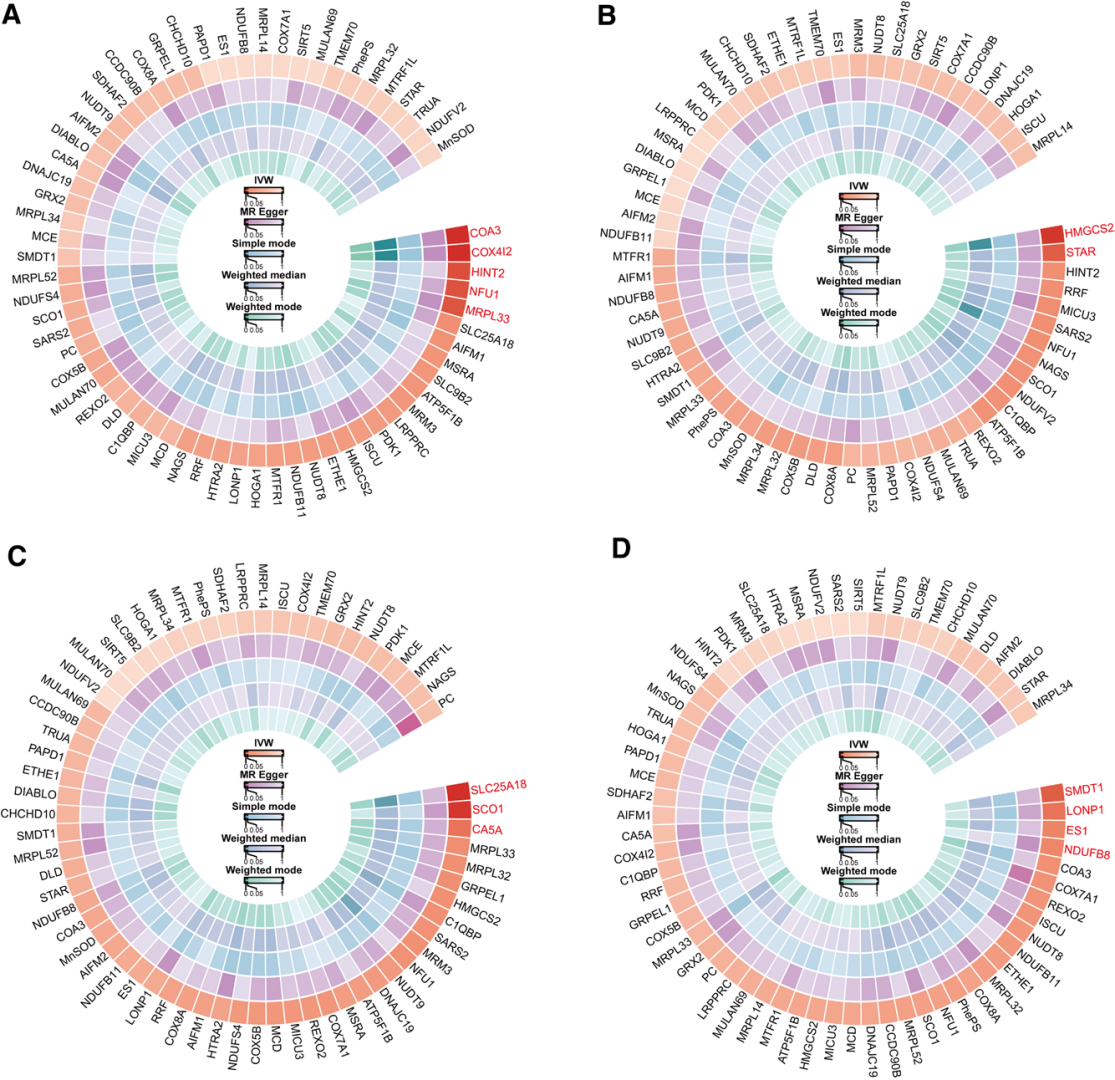
(A) Circular analysis of mitochondrial functional proteins and osteoarthritis association: Highlight the positive results of the IVW method. (B) Circular analysis of mitochondrial functional proteins and osteoporosis association. (C) Circular analysis of mitochondrial functional proteins and rheumatoid arthritis association. (D) Circular analysis of mitochondrial functional proteins and ankylosing spondylitis association. IVW = inverse variance weighting.

We utilized data from the IEU OpenGWAS database, including 66 exposures related to mitochondrial proteins, and musculoskeletal disease data from the UK Biobank database. After strict SNP inclusion and repeated outlier screening, we employed 5 different Mendelian randomization methods: IVW, MR-Egger, simple mode, weighted median, and weighted mode to assess the potential causal interaction between mitochondrial function and musculoskeletal diseases (Fig. 2), with the IVW method as the main result.

We observed that 5 mitochondrial-associated proteins demonstrated close associations with OA (Fig. 2A)^[37]^: COA3 (cytochrome c oxidase assembly factor 3 homolog, mitochondrial), COX4I2 (cytochrome c oxidase subunit 4 isoform 2, mitochondrial), HINT2 (histidine triad nucleotide-binding protein 2, mitochondrial), NFU1 (NFU1 iron–sulfur cluster scaffold homolog, mitochondrial), and MRPL33 (39S ribosomal protein L33, mitochondrial).

In addition, 2 mitochondrial-associated proteins showed a strong association with OP (Fig. 2B): pHMGCS2 (hydroxymethylglutaryl-CoA synthase, mitochondrial) and STAR (steroidogenic acute regulatory protein, mitochondrial).

Three SNP datasets showed high correlation with RA (Fig. 2C): SLC25A18 (mitochondrial glutamate carrier 2), SCO1 (protein SCO1 homolog, mitochondrial), and CA5A (carbonic anhydrase 5A, mitochondrial).

Additionally, 4 SNP datasets are closely related to AS (Fig. 2D): SMDT1 (essential MCU regulator, mitochondrial), LONP1 (Lon protease homolog, mitochondrial) (Fig. 2D), ES1 (ES1 protein homolog, mitochondrial), and NDUFB8 (NADH dehydrogenase [ubiquinone] 1 beta subcomplex subunit 8, mitochondrial).

**Figure 3.**
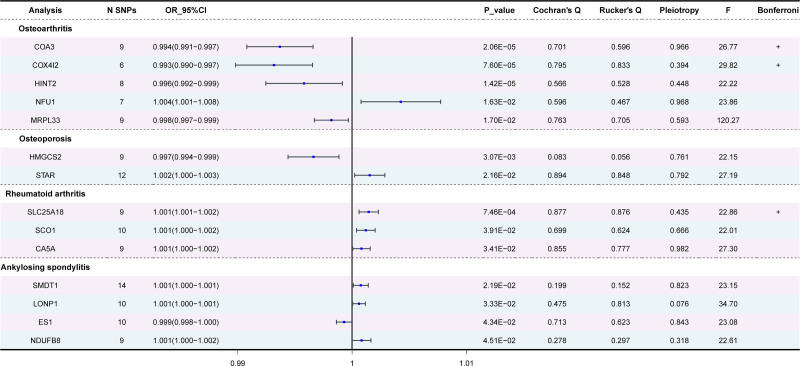
Forest plot of MR analysis on the impact of mitochondrial-related proteins on musculoskeletal diseases. MR = Mendelian randomization.

A total of 14 mitochondria-related datasets were screened. The detailed analysis results are shown in Table S2, Supplemental Digital Content, http://links.lww.com/MD/O462 (Fig. 3).

### 3.2. Sensitivity analysis

To figure out if heterogeneity exists, we employed the MR-Egger method and IVW. The *P* value for both Cochran *Q* and Rucker *Q* statistics > .05 was found in the results for the positive dataset of mitochondrial-associated protein, indicating that heterogeneity was not significant. Subsequently, MR-PRESSO and Egger regression were utilized to investigate the presence of directional horizontal pleiotropy, with all dataset results showing *P* > .05, indicating no evidence of horizontal directional pleiotropy (Fig. 3).

We then performed Bonferroni correction, and the results of COA3 and COX4I2 in the OA dataset and SLC25A18 in the RA dataset remained stable (Fig. 3). For the stability of the results, we also used different methods to verify the causal association for these 3 datasets, and the results were consistent with the IVW direction and were statistically significant (Figure S1, Supplemental Digital Content, http://links.lww.com/MD/O462).

The funnel plot highlighted the relative stability of the results by showing a symmetric distribution of SNPs. We utilized forest plots to illustrate the connection between each genetic variant and disease occurrence. Furthermore, to assess the impact of individual SNPs, we used leave-one-out analysis, which showed no significant impact on the effect size. Figures S2–S4, Supplemental Digital Content, http://links.lww.com/MD/O462 show the results for 3 mitochondrial proteins.

## 4. Discussion

The purpose of this study was to examine the causal relationship between mitochondria and musculoskeletal diseases through Mendelian randomization. After Bonferroni correction, COA3 (*P* < .0001, OR = 0.994, 95% CI = 0.991 − 0.997) and COX4I2 (*P* < .0001, OR = 0.993, 95% CI = 0.990 − 0.997) were identified as protective factors for OA. SLC25A18 was validated as a risk factor for RA (*P* < .0001, OR = 1.001, 95% CI = 1.001 − 1.002). The optimal function of mitochondria is closely associated with musculoskeletal health.^[38]^ Exploring these potentially protective or pathogenic proteins provides new insights into targeted therapeutic intervention and drug advancement in musculoskeletal diseases.

### 4.1. COA3 and OA

Mitochondrial homeostasis is crucial for maintaining cartilage integrity and is linked to the progression of OA.^[39]^ COA3, also known as CCDC56, is a mitochondrial transmembrane protein that participates in the assembly of cytochrome c oxidase (COX) protein complexes.^[40,41]^ The COX complex IV serves as the final enzyme of the mitochondrial respiratory chain, facilitating the synthesis of ATP by moving electrons from reduced cytochrome c to oxygen.^[42]^ hCOA3 can stabilize COX1 and promote the assembly of COX in human mitochondria, thereby promoting cellular ATP production.^[43]^ Previous studies have found that the expression level of COA3 protein is related to lung cancer and can predict the probability of lymph node metastasis.^[40]^ Currently, there are few studies on COA3 in OA or chondrocytes, making it a promising direction for further investigation in the future.

### 4.2. COX4I2 and OA

The COX4 subunit isoform COX4I2 is crucial for maintaining cartilage integrity. Articular chondrocytes may usually survive and keep healthy in avascular and hypoxic environments. However, the consumption of energy and the accumulation of free radicals caused by mitochondrial damage work in concert to initiate and quicken the development of OA.^[44]^ In our study, COX4I2 was a protective factor against OA. Previous studies have demonstrated that reprogramming the SIRT3-COX4I2 axis can reduce the progression of osteoarthritis.^[45]^ Studies have revealed that hypoxia-inducible factor-1 controls the expression of COX4 subunits by triggering the transcription of COX4I2, thus manipulating the activity of the COX family, ATP synthesis, and reactive oxygen species generation. Deficiency of COX4I2 results in combined respiratory chain defects, such as loss of complex I, III, and IV assembly, and reduced respiratory capability or energy supply.^[46]^ This in turn accelerates cartilage extracellular matrix destruction and OA progression. The findings highlight the significant role of COX4I2 in maintaining mitochondrial homeostasis. COX4I2, as a new marker, can promote angiogenesis and epithelial–mesenchymal transition and has been widely discussed in pheochromocytoma, colorectal cancer, and respiratory diseases.^[47–50]^ However, the specific mode of action of COX4I2 in chondrocytes remains to be elucidated.

### 4.3. SLC25A18 and RA

SLC25A18 is a member of the SLC25A family^[51,52]^ which constitutes the mitochondrial carrier system.^[53]^ SLC25A18 facilitates the movement of nutrients from the cytoplasm to mitochondria and has an impact on metabolic reactions and crucial biological processes, including maintaining ion balance, regulating mitochondrial activity, promoting cell differentiation, and regulating cell death.^[54,55]^ SLC25A18 is highly expressed in the brain, liver, and testis.^[56]^ Its ability to facilitate the transportation of glutamate across the inner membrane of mitochondria may contribute to the development of several disorders, including Cat Eye Syndrome, Cutis laxa, and juvenile-onset diabetes. Mutated SLC25A18 is the cause of the autosomal recessive disease citrullinemia.^[57,58]^ Unfortunately, research on SLC25A18 in musculoskeletal disorders is still insufficient and requires further investigation.

This study conducted by Mendelian randomization offers several advantages. Firstly, the findings of Mendelian randomization indicate that 3 proteins exert influence on the development of musculoskeletal diseases, while another 11 proteins exhibit prospective impacts. This offers direction for future investigations into mechanisms. Furthermore, our study employed a substantial quantity of GWAS data samples. By implementing rigorous rectification and repeated screening processes, employing numerous MR verification methods, and performing sensitivity analysis, we enhanced the reliability of the research findings and minimized potential errors. Nevertheless, our study also has some limitations. Firstly, it should be noted that the findings of the GWAS may not be applicable to other ethnic groups due to the fact that all people were of European ancestry. Additionally, it should be mentioned that the primary objective of MR is to make assessments regarding causality rather than measure the magnitude of the effect size. The MR results exhibit a small effect size. It is vital to replicate the data using a larger sample size in order to strengthen the statistic. Furthermore, the quantity of IVs of SNPs in each dataset was comparatively limited. Hence, it is possible that this explanation accounts for a limited amount of the variability in exposure and could potentially impact the statistical robustness of causal estimations.

In conclusion, our study found that COA3 and COX4I2 may be protective factors for OA, and SLC25A18 may be associated with the development of RA. However, additional basic research is required to validate and enhance our findings, which may provide new ideas for the prevention and treatment of musculoskeletal diseases.

## Acknowledgments

We thank all the participants in this study. We also thank the IUE summary database for providing data for the analyses.

## Author contributions

**Conceptualization:** Jia-Chen Li, Qi-Fei He.

**Data curation:** Jia-Chen Li, Wei-Sheng Huang.

**Formal analysis:** Wei-Sheng Huang.

**Funding acquisition:** Wei Sun.

**Investigation:** Jia-Chen Li, Da-Hang Yang.

**Methodology:** Jia-Chen Li.

**Resources:** Da-Hang Yang, Qi-Fei He.

**Supervision:** Wei Sun, Qi-Fei He.

**Writing – review & editing:** Da-Hang Yang, Qi-Fei He.

## Supplementary Material
