# Supplementary material for: Assessing causality between mitochondrial-associated proteins with musculoskeletal diseases: A Mendelian randomization study
Source: Medicine (Baltimore). 2025 Mar 7;104(10):e41731. doi: 10.1097/MD.0000000000041731 (PMC11903026; doi:10.1097/MD.0000000000041731)

Supplementary table 1:GWAS data related to mitochondrial biological functions.

GWAS ID	Year	Trait	Consortium	Sample size	Number of SNPs	Population	Author	PMID	Abbreviation
prot-a-1055	2018	Phenylalanine--tRNA ligase, mitochondrial	INTERVAL	3,301	10,534,735	European	Sun BB	29875488	PhePS
prot-a-1220	2018	Glutaredoxin-2, mitochondrial	INTERVAL	3,301	10,534,735	European	Sun BB	29875488	GRX2
prot-a-1281	2018	GrpE protein homolog 1, mitochondrial	INTERVAL	3,301	10,534,735	European	Sun BB	29875488	GRPEL1
prot-a-1339	2018	Histidine triad nucleotide-binding protein 2,	INTERVAL	3,301	10,534,735	European	Sun BB	29875488	HINT2

		mitochondrial							
prot-a-1356	2018	Hydroxymethylglutaryl-CoA synthase, mitochondrial	INTERVAL	3,301	10,534,735	European	Sun BB	29875488	HMGCS2
prot-a-1368	2018	4-hydroxy-2-oxoglutarate aldolase, mitochondrial	INTERVAL	3,301	10,534,735	European	Sun BB	29875488	HOGA1
prot-a-1392	2018	Serine protease HTRA2, mitochondrial	INTERVAL	3,301	10,534,735	European	Sun BB	29875488	HTRA2
prot-a-1572	2018	Iron-sulfur cluster assembly enzyme	INTERVAL	3,301	10,534,735	European	Sun BB	29875488	ISCU

		ISCU, mitochondrial							
prot-a-1761	2018	Lon protease homolog, mitochondrial	INTERVAL	3,301	10,534,735	European	Sun BB	29875488	LONP1
prot-a-1783	2018	Leucine-rich PPR motif-containing protein, mitochondrial	INTERVAL	3,301	10,534,735	European	Sun BB	29875488	LRPPRC
prot-a-1864	2018	Methylmalonyl-CoA epimerase, mitochondrial	INTERVAL	3,301	10,534,735	European	Sun BB	29875488	MCE
prot-a-1907	2018	Malonyl-CoA decarboxylas	INTERVAL	3,301	10,534,735	European	Sun BB	29875488	MCD

		e, mitochondrial							
prot-a-1940	2018	39S ribosomal protein L14, mitochondrial	INTERVAL	3,301	10,534,735	European	Sun BB	29875488	MRPL14
prot-a-1941	2018	39S ribosomal protein L32, mitochondrial	INTERVAL	3,301	10,534,735	European	Sun BB	29875488	MRPL32
prot-a-1942	2018	39S ribosomal protein L33, mitochondrial	INTERVAL	3,301	10,534,735	European	Sun BB	29875488	MRPL33
prot-a-1943	2018	39S ribosomal protein L34,	INTERVAL	3,301	10,534,735	European	Sun BB	29875488	MRPL34

		mitochondrial							
prot-a-1944	2018	39S ribosomal protein L52, mitochondrial	INTERVAL	3,301	10,534,735	European	Sun BB	29875488	MRPL52
prot-a-1945	2018	Ribosome-recycling factor, mitochondrial	INTERVAL	3,301	10,534,735	European	Sun BB	29875488	RRF
prot-a-1953	2018	Mitochondrial peptide methionine sulfoxide reductase	INTERVAL	3,301	10,534,735	European	Sun BB	29875488	MSRA
prot-a-1961	2018	Mitochondrial fission regulator 1	INTERVAL	3,301	10,534,735	European	Sun BB	29875488	MTFR1

prot-a-1964	2018	Poly(A) RNA polymerase, mitochondrial	INTERVAL	3,301	10,534,735	European	Sun BB	29875488	PAPD1
prot-a-1965	2018	Peptide chain release factor 1-like, mitochondrial	INTERVAL	3,301	10,534,735	European	Sun BB	29875488	MTRF1L
prot-a-1969	2018	Mitochondrial ubiquitin ligase activator of NFKB 1-1969	INTERVAL	3,301	10,534,735	European	Sun BB	29875488	MULAN69
prot-a-1970	2018	Mitochondrial ubiquitin ligase activator of NFKB	INTERVAL	3,301	10,534,735	European	Sun BB	29875488	MULAN70

		1-1970							
prot-a-1997	2018	N-acetylglutamate synthase, mitochondrial	INTERVAL	3,301	10,534,735	European	Sun BB	29875488	NAGS
prot-a-2022	2018	NADH dehydrogenase [ubiquinone] 1 beta subcomplex subunit 11, mitochondrial	INTERVAL	3,301	10,534,735	European	Sun BB	29875488	NDUFB11
prot-a-2024	2018	NADH dehydrogenase [ubiquinone] 1 beta subcomplex subunit 8, mitochondria	INTERVAL	3,301	10,534,735	European	Sun BB	29875488	NDUFB8

		l							
prot-a-2025	2018	NADH dehydrogenase [ubiquinone] iron-sulfur protein 4, mitochondrial	INTERVAL	3,301	10,534,735	European	Sun BB	29875488	NDUFS4
prot-a-2026	2018	NADH dehydrogenase [ubiquinone] flavoprotein 2, mitochondrial	INTERVAL	3,301	10,534,735	European	Sun BB	29875488	NDUFV2
prot-a-203	2018	ATP synthase subunit beta, mitochondrial	INTERVAL	3,301	10,534,735	European	Sun BB	29875488	ATP5F1B

prot-a-2041	2018	NFU1 iron-sulfur cluster scaffold homolog, mitochondrial	INTERVAL	3,301	10,534,735	European	Sun BB	29875488	NFU1
prot-a-2128	2018	Nucleoside diphosphate-linked moiety X motif 8, mitochondrial	INTERVAL	3,301	10,534,735	European	Sun BB	29875488	NUDT8
prot-a-2129	2018	ADP-ribose pyrophosphatase, mitochondrial	INTERVAL	3,301	10,534,735	European	Sun BB	29875488	NUDT9
prot-a-2190	2018	Pyruvate carboxylase, mitochondria	INTERVAL	3,301	10,534,735	European	Sun BB	29875488	PC

		l							
prot-a-2235	2018	[Pyruvate dehydrogenase (acetyl-transferring)] kinase isozyme 1, mitochondrial	INTERVAL	3,301	10,534,735	European	Sun BB	29875488	PDK1
prot-a-2236	2018	[Pyruvate dehydrogenase (acetyl-transferring)] kinase isozyme 2, mitochondrial	INTERVAL	3,301	10,534,735	European	Sun BB	29875488	PDK2
prot-a-2454	2018	tRNA pseudouridine synthase A, mitochondria	INTERVAL	3,301	10,534,735	European	Sun BB	29875488	TRUA

		l							
prot-a-2526	2018	Oligoribonuclease, mitochondrial	INTERVAL	3,301	10,534,735	European	Sun BB	29875488	REXO2
prot-a-2575	2018	rRNA methyltransferase 3, mitochondrial	INTERVAL	3,301	10,534,735	European	Sun BB	29875488	MRM3
prot-a-2627	2018	Serine--tRNA ligase, mitochondrial	INTERVAL	3,301	10,534,735	European	Sun BB	29875488	SARS2
prot-a-2653	2018	Protein SCO1 homolog, mitochondrial	INTERVAL	3,301	10,534,735	European	Sun BB	29875488	SCO1

prot-a-2657	2018	Succinate dehydrogenase assembly factor 2, mitochondrial	INTERVAL	3,301	10,534,735	European	Sun BB	29875488	SDHAF2
prot-a-2737	2018	NAD-dependent protein deacylase sirtuin-5, mitochondrial	INTERVAL	3,301	10,534,735	European	Sun BB	29875488	SIRT5
prot-a-2749	2018	Mitochondrial glutamate carrier 2	INTERVAL	3,301	10,534,735	European	Sun BB	29875488	SLC25A18
prot-a-2764	2018	Mitochondrial sodium/hydrogen exchanger 9B2	INTERVAL	3,301	10,534,735	European	Sun BB	29875488	SLC9B2

prot-a-2776	2018	Essential MCU regulator, mitochondrial	INTERVAL	3,301	10,534,735	European	Sun BB	29875488	SMDT1
prot-a-2799	2018	Superoxide dismutase [Mn], mitochondrial	INTERVAL	3,301	10,534,735	European	Sun BB	29875488	MnSOD
prot-a-2866	2018	Steroidogenic acute regulatory protein, mitochondrial	INTERVAL	3,301	10,534,735	European	Sun BB	29875488	STAR
prot-a-300	2018	Complement component 1 Q subcomponent-binding protein,	INTERVAL	3,301	10,534,735	European	Sun BB	29875488	C1QBP

		mitochondrial							
prot-a-3015	2018	Transmembrane protein 70, mitochondrial	INTERVAL	3,301	10,534,735	European	Sun BB	29875488	TMEM70
prot-a-308	2018	ES1 protein homolog, mitochondrial	INTERVAL	3,301	10,534,735	European	Sun BB	29875488	ES1
prot-a-332	2018	Carbonic anhydrase 5A, mitochondrial	INTERVAL	3,301	10,534,735	European	Sun BB	29875488	CA5A
prot-a-385	2018	Coiled-coil domain-containing protein 90B, mitochondria	INTERVAL	3,301	10,534,735	European	Sun BB	29875488	CCDC90B

		l							
prot-a-534	2018	Coiled-coil-helix-coiled-coil-helix domain-containing protein 10, mitochondrial	INTERVAL	3,301	10,534,735	European	Sun BB	29875488	CHCHD10
prot-a-612	2018	Cytochrome c oxidase assembly factor 3 homolog, mitochondrial	INTERVAL	3,301	10,534,735	European	Sun BB	29875488	COA3
prot-a-63	2018	Apoptosis-inducing factor 1-63, mitochondrial	INTERVAL	3,301	10,534,735	European	Sun BB	29875488	AIFM

prot-a-637	2018	Cytochrome c oxidase subunit 4 isoform 2, mitochondrial	INTERVAL	3,301	10,534,735	European	Sun BB	29875488	COX4I2
prot-a-638	2018	Cytochrome c oxidase subunit 5B, mitochondrial	INTERVAL	3,301	10,534,735	European	Sun BB	29875488	COX5B
prot-a-64	2018	Apoptosis-inducing factor 1-64, mitochondrial	INTERVAL	3,301	10,534,735	European	Sun BB	29875488	AIFM1
prot-a-640	2018	Cytochrome c oxidase subunit 7A1, mitochondrial	INTERVAL	3,301	10,534,735	European	Sun BB	29875488	COX7A1

prot-a-641	2018	Cytochrome c oxidase subunit 8A, mitochondrial	INTERVAL	3,301	10,534,735	European	Sun BB	29875488	COX8A
prot-a-818	2018	Diablo homolog, mitochondrial	INTERVAL	3,301	10,534,735	European	Sun BB	29875488	DIABLO
prot-a-825	2018	Dihydrolipoyl dehydrogenase, mitochondrial	INTERVAL	3,301	10,534,735	European	Sun BB	29875488	DLD
prot-a-847	2018	Mitochondrial import inner membrane translocase subunit TIM14	INTERVAL	3,301	10,534,735	European	Sun BB	29875488	DNAJC19

prot-a-896	2018	Calcium uptake protein 3, mitochondrial	INTERVAL	3,301	10,534,735	European	Sun BB	29875488	MICU3
prot-a-992	2018	Persulfide dioxygenase ETHE1, mitochondrial	INTERVAL	3,301	10,534,735	European	Sun BB	29875488	ETHE1

Supplementary table 2:The detailed MR analysis results.

id.exposure	id.outcome	protein name	methods	nsnps	b	se	P_value	or
prot-a-1942	ukb-b-14486	39S ribosomal protein L33, mitochondrial	Inverse variance weighted	9	-0.001813002	0.000759454	0.016975226	0.998188641
prot-a-1942	ukb-b-14486	39S ribosomal protein L33, mitochondrial	MR Egger	9	-0.002231041	0.001064398	0.074302375	0.997771446
prot-a-1942	ukb-b-14486	39S ribosomal protein L33, mitochondrial	Weighted median	9	-0.001073973	0.001074722	0.317648014	0.998926604
prot-a-1942	ukb-b-14486	39S ribosomal protein L33, mitochondrial	Simple mode	9	-0.001036065	0.001860314	0.592809682	0.998964472
prot-a-1942	ukb-b-14486	39S ribosomal protein L33, mitochondrial	Weighted mode	9	-0.001852227	0.000988212	0.097755494	0.998149487
prot-a-612	ukb-b-14486	Cytochrome c oxidase assembly factor 3 homolog, mitochondrial	Inverse variance weighted	9	-0.006330524	0.00148662	2.05957E-05	0.993689472

prot-a-612	ukb-b-14486	Cytochrome c oxidase assembly factor 3 homolog, mitochondrial	MR Egger	9	-0.006435979	0.002813277	0.055995731	0.993584687
prot-a-612	ukb-b-14486	Cytochrome c oxidase assembly factor 3 homolog, mitochondrial	Weighted median	9	-0.006291841	0.002023337	0.001873188	0.993727912
prot-a-612	ukb-b-14486	Cytochrome c oxidase assembly factor 3 homolog, mitochondrial	Simple mode	9	-0.006921052	0.00333151	0.071397394	0.993102843
prot-a-612	ukb-b-14486	Cytochrome c oxidase assembly factor 3 homolog, mitochondrial	Weighted mode	9	-0.007658694	0.003204477	0.043855965	0.992370559
prot-a-637	ukb-b-14486	Cytochrome c oxidase subunit 4 isoform 2, mitochondrial	Inverse variance weighted	6	-0.006852437	0.001731849	7.5987E-05	0.993170987
prot-a-637	ukb-b-14486	Cytochrome c oxidase subunit 4 isoform 2, mitochondrial	MR Egger	6	-0.009995069	0.00372416	0.055008414	0.990054716

prot-a-637	ukb-b-14486	Cytochrome c oxidase subunit 4 isoform 2, mitochondrial	Weighted median	6	-0.007566047	0.002295005	0.000978123	0.992462503
prot-a-637	ukb-b-14486	Cytochrome c oxidase subunit 4 isoform 2, mitochondrial	Simple mode	6	-0.008487026	0.003615527	0.065773722	0.991548887
prot-a-637	ukb-b-14486	Cytochrome c oxidase subunit 4 isoform 2, mitochondrial	Weighted mode	6	-0.008559882	0.003154466	0.042096218	0.99147665
prot-a-1339	ukb-b-14486	Histidine triad nucleotide-binding protein 2, mitochondrial	Inverse variance weighted	8	-0.004210117	0.001716676	0.014187465	0.995798733
prot-a-1339	ukb-b-14486	Histidine triad nucleotide-binding protein 2, mitochondrial	MR Egger	8	-0.001165288	0.004126352	0.78712435	0.99883539
prot-a-1339	ukb-b-14486	Histidine triad nucleotide-binding protein 2, mitochondrial	Weighted median	8	-0.003192041	0.002342024	0.172901137	0.996813049

prot-a-1339	ukb-b-14486	Histidine triad nucleotide-binding protein 2, mitochondrial	Simple mode	8	-0.001797469	0.003814	0.651774049	0.998204145
prot-a-1339	ukb-b-14486	Histidine triad nucleotide-binding protein 2, mitochondrial	Weighted mode	8	-0.00174556	0.003613281	0.643770917	0.998255962
prot-a-2041	ukb-b-14486	NFU1 iron-sulfur cluster scaffold homolog, mitochondrial	Inverse variance weighted	7	0.004250302	0.001769934	0.016332891	1.004259347
prot-a-2041	ukb-b-14486	NFU1 iron-sulfur cluster scaffold homolog, mitochondrial	MR Egger	7	0.004085633	0.00431571	0.387268238	1.00409399
prot-a-2041	ukb-b-14486	NFU1 iron-sulfur cluster scaffold homolog, mitochondrial	Weighted median	7	0.002777483	0.002355347	0.238308744	1.002781344
prot-a-2041	ukb-b-14486	NFU1 iron-sulfur cluster scaffold homolog, mitochondrial	Simple mode	7	0.001070844	0.004052282	0.800424588	1.001071417

prot-a-2041	ukb-b-14486	NFU1 iron-sulfur cluster scaffold homolog, mitochondrial	Weighted mode	7	0.001023522	0.003605841	0.786065138	1.001024046
prot-a-1356	ukb-a-87	Hydroxymethylglutaryl-CoA synthase, mitochondrial	Inverse variance weighted	9	-0.003379753	0.001141633	0.003071848	0.996625952
prot-a-1356	ukb-a-87	Hydroxymethylglutaryl-CoA synthase, mitochondrial	MR Egger	9	-0.00269937	0.002468274	0.310318448	0.99730427
prot-a-1356	ukb-a-87	Hydroxymethylglutaryl-CoA synthase, mitochondrial	Weighted median	9	-0.00338845	0.001314617	0.009951252	0.996617284
prot-a-1356	ukb-a-87	Hydroxymethylglutaryl-CoA synthase, mitochondrial	Simple mode	9	-0.005491605	0.002734248	0.079468681	0.994523447
prot-a-1356	ukb-a-87	Hydroxymethylglutaryl-CoA synthase, mitochondrial	Weighted mode	9	-0.005043723	0.002552322	0.083547416	0.994968975
prot-a-2866	ukb-a-87	Steroidogenic acute regulatory protein, mitochondrial	Inverse variance weighted	12	0.001544106	0.000672107	0.021595303	1.001545299
prot-a-2	ukb-a-	Steroidogenic acute	MR Egger	12	0.001946976	0.001631275	0.260207576	1.001948873

866	87	regulatory protein, mitochondrial						
prot-a-2 866	ukb-a-87	Steroidogenic acute regulatory protein, mitochondrial	Weighted median	12	0.001258727	0.000872551	0.149137814	1.00125952
prot-a-2 866	ukb-a-87	Steroidogenic acute regulatory protein, mitochondrial	Simple mode	12	0.001262927	0.001329434	0.362530992	1.001263725
prot-a-2 866	ukb-a-87	Steroidogenic acute regulatory protein, mitochondrial	Weighted mode	12	0.001166046	0.001283756	0.383184413	1.001166726
prot-a-2 653	ukb-d-M06	Protein SCO1 homolog, mitochondrial	Inverse variance weighted	10	0.001198145	0.000415291	0.003913191	1.001198863
prot-a-2 653	ukb-d-M06	Protein SCO1 homolog, mitochondrial	MR Egger	10	0.001750507	0.001300965	0.215338454	1.00175204
prot-a-2 653	ukb-d-M06	Protein SCO1 homolog, mitochondrial	Weighted median	10	0.00089664	0.000580104	0.122188434	1.000897042

prot-a-2653	ukb-d-M06	Protein SCO1 homolog, mitochondrial	Simple mode	10	0.000371888	0.000896349	0.687936071	1.000371957
prot-a-2653	ukb-d-M06	Protein SCO1 homolog, mitochondrial	Weighted mode	10	0.000345113	0.000966202	0.729185513	1.000345172
prot-a-2749	ukb-d-M06	Mitochondrial glutamate carrier 2	Inverse variance weighted	9	0.001452066	0.000430611	0.000745945	1.001453121
prot-a-2749	ukb-d-M06	Mitochondrial glutamate carrier 2	MR Egger	9	0.000659106	0.001050743	0.550382761	1.000659323
prot-a-2749	ukb-d-M06	Mitochondrial glutamate carrier 2	Weighted median	9	0.001351194	0.000584233	0.020735622	1.001352107
prot-a-2749	ukb-d-M06	Mitochondrial glutamate carrier 2	Simple mode	9	0.001371349	0.000889242	0.16160817	1.00137229
prot-a-2749	ukb-d-M06	Mitochondrial glutamate carrier 2	Weighted mode	9	0.001371349	0.000878474	0.157132872	1.00137229
prot-a-332	ukb-d-M06	Carbonic anhydrase 5A, mitochondrial	Inverse variance weighted	9	0.000819513	0.000386808	0.034119556	1.000819849

prot-a-332	ukb-d-M06	Carbonic anhydrase 5A, mitochondrial	MR Egger	9	0.00084219	0.001037442	0.443634058	1.000842545
prot-a-332	ukb-d-M06	Carbonic anhydrase 5A, mitochondrial	Weighted median	9	0.000932272	0.000506803	0.065839664	1.000932707
prot-a-332	ukb-d-M06	Carbonic anhydrase 5A, mitochondrial	Simple mode	9	0.000821829	0.000745437	0.302311673	1.000822167
prot-a-332	ukb-d-M06	Carbonic anhydrase 5A, mitochondrial	Weighted mode	9	0.00106909	0.000748969	0.191305959	1.001069662
prot-a-1761	ukb-a-88	Lon protease homolog, mitochondrial	Inverse variance weighted	10	0.000608067	0.000285727	0.033325335	1.000608251
prot-a-1761	ukb-a-88	Lon protease homolog, mitochondrial	MR Egger	10	-0.00079981	0.000748788	0.316626183	0.99920051
prot-a-1761	ukb-a-88	Lon protease homolog, mitochondrial	Simple mode	10	0.001085992	0.000757867	0.18567716	1.001086582
prot-a-1761	ukb-a-88	Lon protease homolog, mitochondrial	Weighted median	10	0.000279749	0.000390589	0.473853182	1.000279789

prot-a-1761	ukb-a-88	Lon protease homolog, mitochondrial	Weighted mode	10	0.000145482	0.000448293	0.752957457	1.000145492
prot-a-2024	ukb-a-88	NADH dehydrogenase [ubiquinone] 1 beta subcomplex subunit 8, mitochondrial	Inverse variance weighted	9	0.000833314	0.000415814	0.04506432	1.000833661
prot-a-2024	ukb-a-88	NADH dehydrogenase [ubiquinone] 1 beta subcomplex subunit 8, mitochondrial	MR Egger	9	0.001726254	0.000926531	0.104728079	1.001727745
prot-a-2024	ukb-a-88	NADH dehydrogenase [ubiquinone] 1 beta subcomplex subunit 8, mitochondrial	Simple mode	9	-0.000205121	0.000964518	0.836905584	0.9997949
prot-a-2024	ukb-a-88	NADH dehydrogenase [ubiquinone] 1 beta subcomplex subunit 8, mitochondrial	Weighted median	9	8.3577E-05	0.000563446	0.882080896	1.000083581
prot-a-2	ukb-a-	NADH dehydrogenase [ubiquinone] 1 beta	Weighted mode	9	-0.000143989	0.000909642	0.878149252	0.999856021

024	88	subcomplex subunit 8, mitochondrial						
prot-a-2776	ukb-a-88	Essential MCU regulator, mitochondrial	Inverse variance weighted	14	0.000763285	0.000332913	0.021862709	1.000763576
prot-a-2776	ukb-a-88	Essential MCU regulator, mitochondrial	MR Egger	14	0.000584167	0.000855405	0.507632574	1.000584338
prot-a-2776	ukb-a-88	Essential MCU regulator, mitochondrial	Simple mode	14	0.001096119	0.000821904	0.205220368	1.00109672
prot-a-2776	ukb-a-88	Essential MCU regulator, mitochondrial	Weighted median	14	0.000577284	0.000428232	0.177638227	1.000577451
prot-a-2776	ukb-a-88	Essential MCU regulator, mitochondrial	Weighted mode	14	0.000389334	0.000751128	0.612935329	1.000389409
prot-a-308	ukb-a-88	ES1 protein homolog, mitochondrial	Inverse variance weighted	10	-0.000695229	0.000344194	0.043395887	0.999305012
prot-a-308	ukb-a-88	ES1 protein homolog, mitochondrial	MR Egger	10	-0.000495428	0.001033962	0.644651818	0.999504694

prot-a-308	ukb-a-88	ES1 protein homolog, mitochondrial	Simple mode	10	-0.000675083	0.000735646	0.382723716	0.999325145
prot-a-308	ukb-a-88	ES1 protein homolog, mitochondrial	Weighted median	10	-0.000615585	0.000460185	0.180997497	0.999384604
prot-a-308	ukb-a-88	ES1 protein homolog, mitochondrial	Weighted mode	10	-0.000675083	0.000679117	0.346166592	0.999325145

Supplementary figure 1:Forest plot of sensitivity analysis result on the impact of positive mitochondrial-related proteins on musculoskeletal diseases.

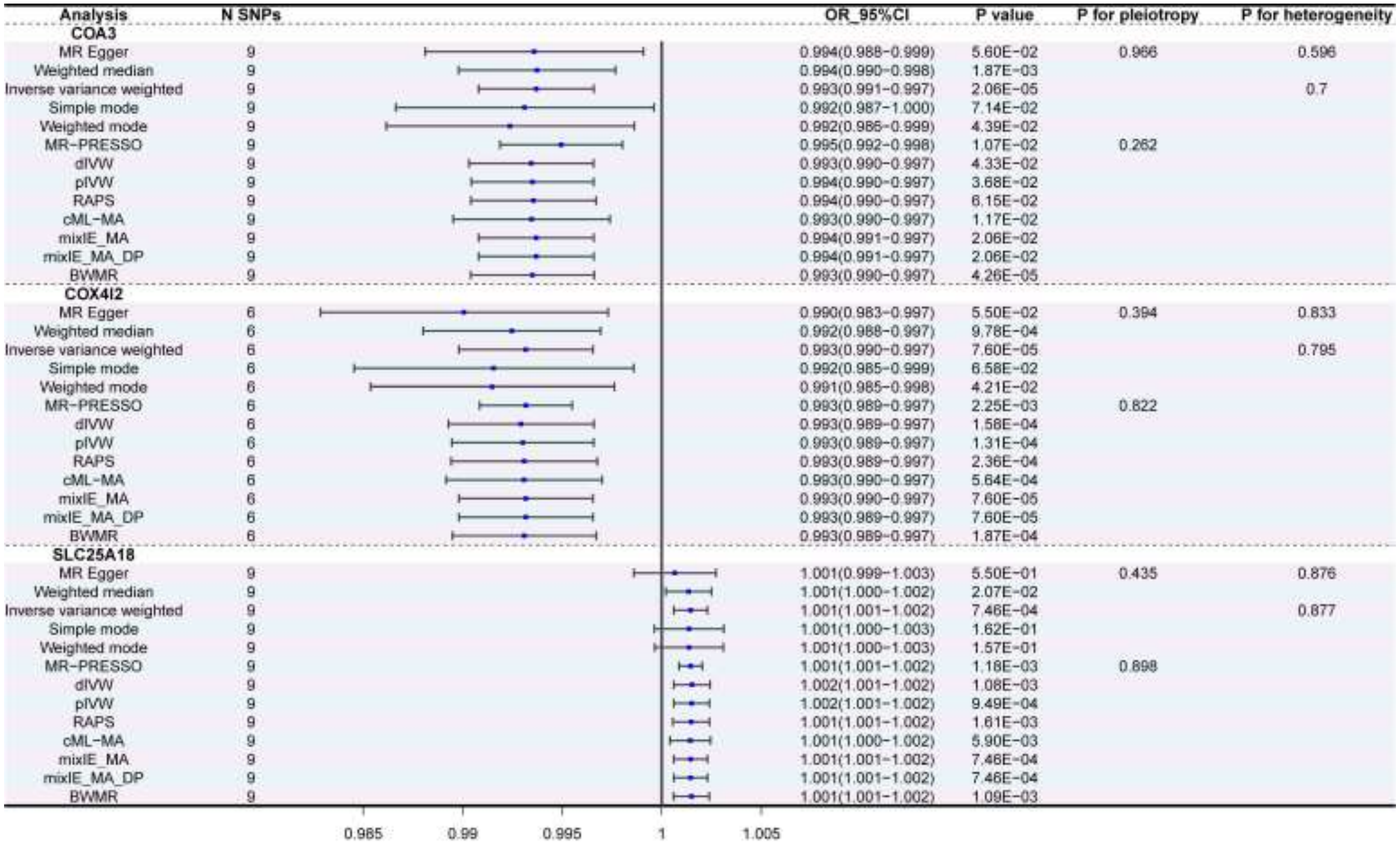

Analysis	N SNPs	OR_95%CI	P value	P for pleiotropy	P for heterogeneity
**COA3**					
MR Egger	9	0.994(0.988−0.999)	5.60E−02	0.966	0.596
Weighted median	9	0.994(0.990−0.998)	1.87E−03		
Inverse variance weighted	9	0.993(0.991−0.997)	2.06E−05		0.7
Simple mode	9	0.992(0.987−1.000)	7.14E−02		
Weighted mode	9	0.992(0.986−0.999)	4.39E−02		
MR−PRESSO	9	0.995(0.992−0.998)	1.07E−02	0.262	
dIVW	9	0.993(0.990−0.997)	4.33E−02		
pIVW	9	0.994(0.990−0.997)	3.68E−02		
RAPS	9	0.994(0.990−0.997)	6.15E−02		
cML−MA	9	0.993(0.990−0.997)	1.17E−02		
mixIE_MA	9	0.994(0.991−0.997)	2.06E−02		
mixIE_MA_DP	9	0.994(0.991−0.997)	2.06E−02		
BWMR	9	0.993(0.990−0.997)	4.26E−05		
**COX4I2**					
MR Egger	6	0.990(0.983−0.997)	5.50E−02	0.394	0.833
Weighted median	6	0.992(0.988−0.997)	9.78E−04		
Inverse variance weighted	6	0.993(0.990−0.997)	7.60E−05		0.795
Simple mode	6	0.992(0.985−0.999)	6.58E−02		
Weighted mode	6	0.991(0.985−0.998)	4.21E−02		
MR−PRESSO	6	0.993(0.989−0.997)	2.25E−03	0.822	
dIVW	6	0.993(0.989−0.997)	1.58E−04		
pIVW	6	0.993(0.989−0.997)	1.31E−04		
RAPS	6	0.993(0.989−0.997)	2.36E−04		
cML−MA	6	0.993(0.990−0.997)	5.64E−04		
mixIE_MA	6	0.993(0.990−0.997)	7.60E−05		
mixIE_MA_DP	6	0.993(0.989−0.997)	7.60E−05		
BWMR	6	0.993(0.989−0.997)	1.87E−04		
**SLC25A18**					
MR Egger	9	1.001(0.999−1.003)	5.50E−01	0.435	0.876
Weighted median	9	1.001(1.000−1.002)	2.07E−02		
Inverse variance weighted	9	1.001(1.001−1.002)	7.46E−04		0.877
Simple mode	9	1.001(1.000−1.003)	1.62E−01		
Weighted mode	9	1.001(1.000−1.003)	1.57E−01		
MR−PRESSO	9	1.001(1.001−1.002)	1.18E−03	0.898	
dIVW	9	1.002(1.001−1.002)	1.08E−03		
pIVW	9	1.002(1.001−1.002)	9.49E−04		
RAPS	9	1.001(1.001−1.002)	1.61E−03		
cML−MA	9	1.001(1.000−1.002)	5.90E−03		
mixIE_MA	9	1.001(1.001−1.002)	7.46E−04		
mixIE_MA_DP	9	1.001(1.001−1.002)	7.46E−04		
BWMR	9	1.001(1.001−1.002)	1.09E−03		

Supplementary figure 2:The results of COA3.

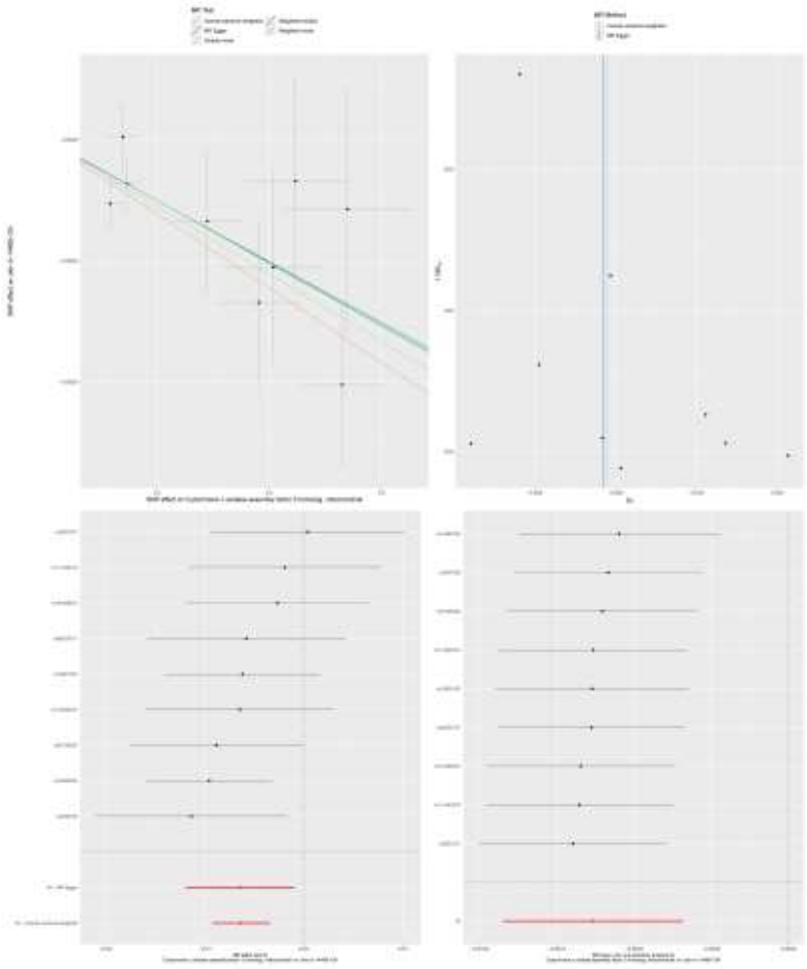

Supplementary figure 3:The results of COX4I2.

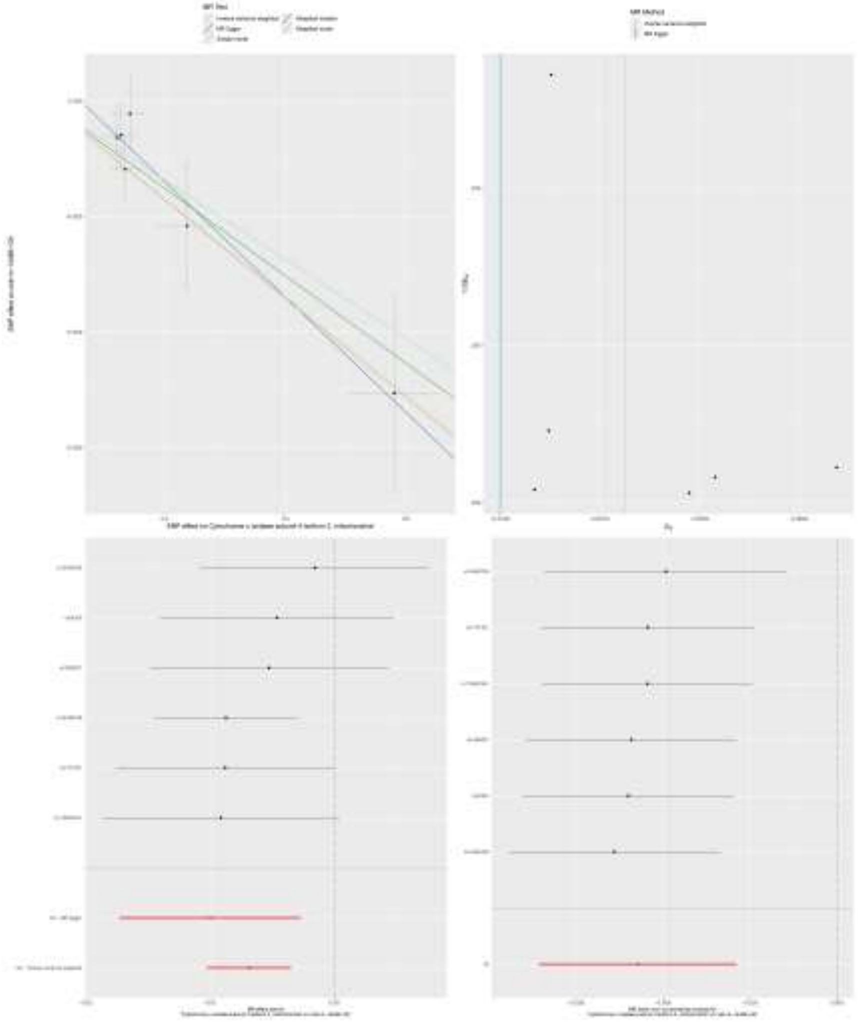

Supplementary figure 4:The results of SLC25A18.

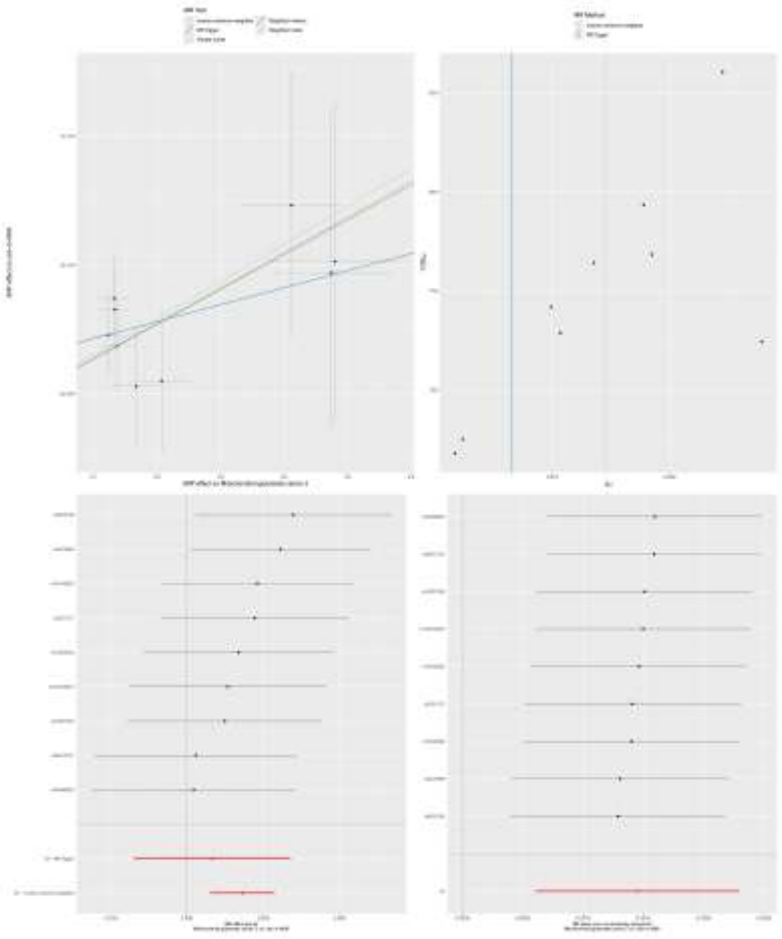

Supplement: Supplementary file 1 [file medi-104-e41731-s001.pdf]
